# Feasibility and acceptability of a cancer symptom awareness intervention for adults living in socioeconomically deprived communities

**DOI:** 10.1186/s12889-018-5606-3

**Published:** 2018-06-05

**Authors:** Pamela Smith, Stephanie Smits, Sioned Owen, Fiona Wood, Grace McCutchan, Ben Carter, Adrian Edwards, Michael Robling, Julia Townson, Kate Brain

**Affiliations:** 1 Division of Population Medicine, Cardiff University, 1st Floor, Neuadd Meirionnydd, Heath Park, Cardiff, CF14 4YS UK; 2 Tenovus Cancer Care, Gleider House, Ty-Glas Rd, Cardiff, CF14 5BD UK; 3 King’s College London, Strand, London, WC2R 2LS UK; 4 Centre for Trials Research, Cardiff University, Heath Park, Cardiff, CF14 4YS UK

**Keywords:** Cancer, Complex intervention, Qualitative, Behaviour change, Inequality, Symptom presentation, Feasibility, Awareness, Socioeconomic deprivation

## Abstract

**Background:**

Cancer survival rates in the UK are lower in comparison with similar countries in Europe and this may be linked to socioeconomic inequalities in stage of cancer diagnosis and survival. Targeted cancer awareness interventions have the potential to improve earlier symptomatic diagnosis and reduce socioeconomic inequalities in cancer outcomes. The health check is an innovative, theory-based intervention designed to increase awareness of cancer symptoms and risk factors, and encourage timely help seeking among adults living in deprived communities.

**Methods:**

A prospective, non-randomised evaluation was undertaken to test the feasibility and acceptability of the health check for adults aged 40 years and over living in deprived areas of Wales. Primary outcomes included recruitment and retention of approximately 100 adults, reach to participants in the lowest deprivation quartile, and intervention acceptability. Secondary outcomes included self-reported cancer symptom recognition, help-seeking behaviours and state anxiety pre/post intervention.

**Results:**

Of 185 individuals approached, 98 (53%) completed the intervention. Sixty-six of 98 participants were recruited from community settings (67%) and 32 from healthcare settings (33%), with 56 (57%) from the lowest deprivation quartile. Eighty-three (85%) participants completed follow-up assessment. Participants recognised on average one extra cancer symptom post intervention, with improved recognition of and anticipated presentation for non-specific symptoms. State anxiety scores remained stable. Qualitative interviews (*n* = 25) demonstrated that the intervention was well received and motivated change.

**Conclusions:**

Recruitment was feasible in community and healthcare settings, with good reach to adults from low socioeconomic groups. The health check intervention was acceptable and demonstrated potential for improved cancer awareness and symptom presentation, especially for non-specific symptoms, in communities most affected by cancer.

**Electronic supplementary material:**

The online version of this article (10.1186/s12889-018-5606-3) contains supplementary material, which is available to authorized users.

## Background

Cancer is the leading cause of deaths in high income countries [1] and research has shown that 42% of people who died in the UK during 2008 had a cancer diagnosis at some point in their life, with tumours being the cause of death in 64% of these patients [2]. Half of people diagnosed with cancer in the UK survive for 10 or more years and this survival rate has doubled over the past 40 years [3]. However, UK survival rates have been consistently lower in comparison with similar countries in Europe [4–7] and this may be linked to socioeconomic inequalities in stage of cancer diagnosis [8–10]. The “patient interval” is defined as the time between appraising a bodily change as a potential symptom of cancer and presenting in primary care [11]. The patient interval accounts for the greatest proportion of time in the pathway from discovering a symptom to the start of cancer treatment [12], and has been found to lengthen with increasing socioeconomic deprivation [13]. The revised NAEDI (National Awareness and Early Diagnosis Initiative) pathway (see Additional file 1) describes the influence of socioeconomic status on cancer survival and premature mortality in the United Kingdom [14]. The NAEDI pathway hypothesises that background factors such as low public awareness, barriers to help-seeking and negative beliefs about cancer can negatively influence presentation to primary care.

Barriers to early cancer symptom presentation include lack of knowledge about potential cancer symptoms [15], fatalism and denial [16], fear of treatment and diagnosis, fear of dying [17] and misinterpretation of symptom seriousness [18]. A systematic review of the influences of awareness and beliefs on symptom presentation demonstrated that fearful and fatalistic beliefs about cancer are associated with longer symptom presentation times in individuals from areas of socioeconomic deprivation [16]. Evidence-based initiatives that aim to increase awareness of potential cancer symptoms and minimise barriers to early cancer diagnosis among people living in deprived communities have the potential to improve cancer outcomes and reduce socioeconomic inequalities [11, 18].

The health check is a theory-based intervention designed and developed in a previous phase of work undertaken by the authors in partnership with Tenovus Cancer Care, a Welsh cancer charity. The aim of the health check is to improve awareness of cancer symptoms and risk factors, encourage positive beliefs about early detection, and increase motivation to seek help among adults living in socioeconomically deprived communities. The intervention is primarily designed to reduce the patient interval, but also includes advice on cancer screening and lifestyle risk factors in order to synergise early detection and prevention messages [19]. To our knowledge this is the first study in the United Kingdom to use an interactive touchscreen tablet as the technological component of a multifaceted, complex intervention aimed at improving cancer awareness and symptom presentation.

In accordance with the MRC framework for complex interventions [20], developmental work to refine the health check was previously undertaken in phase 1 of ABACus (Awareness and Beliefs About Cancer) from November 2014 to October 2015, and was informed by a theoretical understanding of barriers and enablers to timely help seeking among people living in disadvantaged communities [19]. During phase 1, the Behaviour Change Wheel [21] was used to refine the delivery and content of the health check through a systematic process involving the identification of four intervention functions (education,^1^ enablement,^2^ persuasion^3^ and environmental restructuring^4^) and corresponding behaviour change techniques to include in the intervention [22]. Importantly, the health check is facilitated by a lay advisor who is trained to engage participants and deliver theory-based behaviour change techniques.

The purpose of the current study (ABACus phase 2) was to test the feasibility and acceptability of the health check intervention in community and health care settings in socioeconomically deprived areas. Specific objectives were to conduct a before and after questionnaire study to evaluate feasibility of recruitment, reach to and retention of the target audience (primary outcomes), potential for change in cancer symptom recognition and help-seeking intentions/behaviours and unintended consequences relating to anxiety (secondary outcomes), and to carry out qualitative interviews to understand how the intervention was viewed by adults living in deprived communities.

## Methods

The research was conducted in line with the MRC guidance for development and evaluation of complex interventions [20]. The study materials and protocol were approved by NHS Health Research Authority: National Research Ethics Service (Reference: 14/NW/1104) and all participants gave written informed consent.

### The intervention

The health check is a tablet-based interactive touchscreen questionnaire that takes around 30–45 min to complete and is delivered face-to-face by a trained lay advisor. The touchscreen questionnaire includes 26 questions covering the domains of personal history (“About you”), lifestyle (“Your lifestyle”) and symptom experience (“Your health”) (see Additional file 2 for full list of questions). Personalised results are given in a traffic light system, with ‘green’ indicating results where no signposting or change is suggested, ‘amber’ indicating an area where signposting or change could be considered, and ‘red’ results indicating that action should be taken. Categorisation of results for lifestyle risk factors is based on existing NICE, NHS and government guidelines [23–26]. Manualised advice and signposting to relevant services (for example General Practitioner, local stop smoking services and weight loss services) are provided by the lay advisor based on the personalised results. At the end of the health check, participants receive a printout of their results and a brief action plan. The health check content, underpinning intervention functions and behaviour change techniques are detailed in Table 1.

**Table 1:** Health check intervention content

Intervention components	Description of component	Purpose of component	Summary of intervention functions	Behaviour change techniques [22]	Example of application within the intervention
Touchscreen questionnaire: “About you” (7 questions)	Background information about the participant including personal and family history of cancer, body mass index and cancer screening attendance^a^.	Contextual information about potential risk factors for cancer.	Education, persuasion, environmental restructuring	Information about health consequences^b^, prompts/cues^c^	Information about the benefits of early diagnosis. Information about factors that may increase the risk of developing cancer (e.g. being overweight). Questions about previous engagement with cancer screening.
Touchscreen questionnaire: “Your lifestyle” (5 questions)	Diet, smoking, alcohol consumption and physical activity.	Contextual information about potential risk factors for cancer.	Education, persuasion, enablement	Information about health consequences, credible source^d^, social support^e^	Signposting to local services, such as Stop Smoking Wales. Encouragement to pass the information on to friends or family.
Touchscreen questionnaire: “Your health” (14 questions)	Cancer warning signs and symptoms (see Additional file 2)	Contextual information about potential symptoms of cancer.	Education, persuasion, environmental restructuring	Information about health consequences, prompts/cues, credible source	Signposting to General Practitioner. Information about cancer warning signs and symptoms to encourage early presentation within three weeks of noticing a potential symptom (now and in the future).
Personalised results	Displays a printable summary of the individual’s results and action (for example, to present to their General Practitioner with potential cancer symptoms).	Provides participants with an overview of their health check results, to act as a prompt for change (e.g. discussion at their GP appointment).	Education, enablement	Information about health consequences, action planning^f^, goal setting ^g^	Participants complete the following statement: *“If I notice a symptom, I will go and see my ________ within _______ of noticing the symptom*”. Remind participants about the benefits of early diagnosis.

^a^National cancer screening programmes in Wales include bowel (every two years for men and women, aged 60–74), breast (all women aged 50–70) and cervical (women aged 25–49 every three years, women aged 50–64 every five years)

^b^Providing information about health consequences of performing the suggested behaviour

^c^Introduction and definition of environmental or social stimulus with the purpose of prompting or cueing the suggested behaviour

^d^Presenting verbal or visual communication from the credible source in favour of or against a behaviour

^e^Advising on practical and emotional social support (e.g. from friends or family)

^f^Prompt detailed planning of performance of the behaviour (e.g. inclusion of context, frequency, duration and intensity)

^g^Setting or agreeing a goal defined in terms of behaviour to be achieved

### Sample

Participants were adults aged 40 years and over recruited opportunistically in healthcare and non-medical community settings in “Communities First” areas. Communities First was a Welsh Government programme that focused on tackling poverty by supporting the most disadvantaged people in the most deprived areas of Wales. Non-English speakers and those unable to give informed consent were excluded. Participants were approached at each site by a member of the research team and provided with study materials. Participants were offered a £10 high street shopping voucher after completion of the baseline questionnaire and intervention. A further £5 high street shopping voucher was offered at completion of the one month follow-up questionnaire.

The purposive sampling framework consisted of ten settings across two study sites. Participants were recruited from settings identified during phase 1: three community based locations (local community groups, one-to-one sessions, local events) and two healthcare settings (GP practices, community pharmacies). Existing community contacts, such as Communities First staff and community pharmacy managers, facilitated the identification of settings.

### Data collection procedures

Data regarding the number of participants who were approached, agreed to participate, completed the baseline questionnaire, and completed one month follow-up were collected by the researcher (PS). Baseline questionnaire data were collected by PS, who had relevant training in qualitative research methods. The data were captured on computer based forms using an iPad, for direct capture to a secure Cardiff University server. The health check took place in a suitable private room with the lay advisor present. One month follow-up took place by telephone and those who were unable to be contacted after three attempts were sent a postal version of the questionnaire and a pre-paid envelope.

### Socio-demographic characteristics

Socio-demographic characteristics were gathered on age, sex, education level, employment status, ethnic origin, home/living arrangement and relationship status. Socioeconomic group was assessed by matching postcodes to the Welsh Index of Multiple Deprivation (WIMD) (lower super-output areas).

### Outcome measures

#### Primary outcomes

Primary outcomes included recruitment of 100 participants within a four month period, recruitment of at least 50% of participants in the lowest deprivation quartile, and a loss to follow-up rate of no higher than 30%.

#### Secondary outcomes

##### Cancer symptom recognition

Cancer symptom recognition was measured using items adapted from the validated ABC measure [27]. Recognition of potential cancer symptoms was assessed using the question stem ‘*Please tell us if you think the following are warning signs of cancer*’ followed by a series of cancer symptoms. Symptoms included in the ABC were adapted to assess recognition of 14 symptoms that were included in the intervention. Responses were dichotomised for analysis (i.e. ‘yes’ versus ‘no/don’t know’), with ‘yes’ responses summed to derive a total cancer symptom recognition score with a score range of 0 to 14 [27].

##### Anticipated symptom presentation

The ABC measure [27] was adapted to assess anticipated time to presentation for symptoms that could indicate cancer. To reduce participant burden, anticipated presentation was anchored to two classic cancer symptoms (‘*an unusual lump’, ‘blood in your poo*’) and two non-specific symptoms (‘*a cough that won’t go away’, ‘losing weight without trying to*’). Response options were recoded to create a binary measure of anticipated symptom presentation (‘more than 3 weeks’ and ‘under 3 weeks’).

##### State anxiety

The short-form state scale of the State Trait Anxiety Inventory (STAI) [28] was included to measure unintended negative consequences of taking part in the health check.

##### Process evaluation measures

Three questions were included to evaluate intervention acceptability: ‘*How useful did you find the information in the health check?*’ (‘not at all useful’, ‘somewhat useful’, ‘moderately useful’ and ‘very useful’); ‘*What did you think about the amount of information in the health check*?’ (‘not enough’, ‘about right’ and ‘too much’), and ‘*Would you recommend the health check to friends or family?*’ (‘yes’ or ‘no’).

##### Qualitative interviews

The baseline consent form included permission to contact participants regarding further participation in process evaluation interviews after they completed the one month follow-up questionnaire. Participants were sampled using maximum variation sampling based on age, gender and intervention location, and interviewed after completion of one month follow-up. Study recruitment materials were posted to those who expressed an interest and the researcher contacted respondents to arrange a time, date and location to carry out the interview. Participants were also given a £10 shopping voucher after the interview. Face-to-face interviews were conducted by PS and explored use of the health check, views and feedback on acceptability of the health check setting, and perceptions of whether any indicated change in behaviour was acceptable and supported by friends/family members.

##### Analysis

Data regarding recruitment, retention rates and socio demographic characteristics of participants were summarised. Questionnaire data were summarised with an arithmetic mean, and the crude mean change in total cancer symptom recognition score from baseline to one month follow-up was analysed. Where participants failed to respond to individual items within an instrument at a specific time point, but completed more than 75% of items, an arithmetic average was imputed for the items that they failed to answer. Participants who were recorded as having missing instrument data were not included in the analysis population. Descriptive statistics were used to assess proportions of individual cancer symptoms recognised and anticipated time to symptom presentation at baseline and one month. Statistical analysis was carried out using IBM SPSS Statistics V.23.

The anonymised process evaluation interviews were analysed thematically by PS using NVivo. An inductive approach to the data was adopted which involved familiarisation with the data, coding and searching, reviewing and defining themes that emerged. Dual coding of 20% of the interview transcripts was conducted by SS and KB, and discrepancies were resolved through discussion.

## Results

### Primary outcomes

#### Study recruitment

**Fig. 1:**
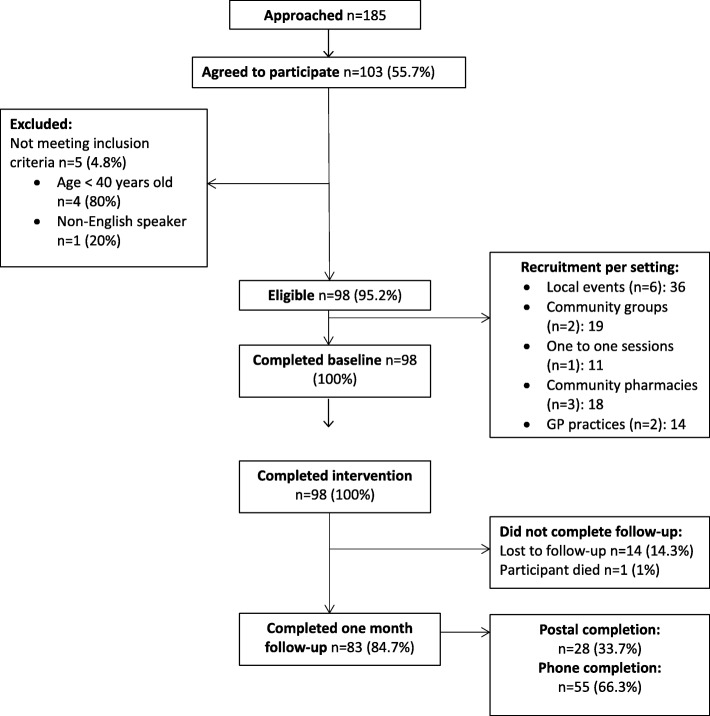
Study recruitment

One hundred and eighty five people were approached to take part in the study and 103 (56%) agreed to participate. Five of those who agreed to take part did not meet the inclusion criteria. A total of 98 people were eligible (95%) and 100% of these completed the baseline questionnaire and intervention. There was a loss to follow-up of 15% with 83 participants (85%) completing the one month follow-up assessment. Sixty-six participants (67%) were recruited in community settings and 32 (33%) were recruited in healthcare settings. Figures demonstrating intervention feasibility, including rates of completion using telephone and postal follow-up methods, are presented in Fig. 1.

#### Socio-demographic characteristics

Baseline socio-demographic characteristics of the 98 respondents are shown in Table 1. Sixty-one percent of participants were aged 60 years or more and 65% were female. Fifty-seven percent of participants were from the lowest WIMD deprivation quartile. Fifty-one percent or participants were retired and 55% had no formal education. Ninety-three percent of the participants described their ethnic origin as British and 12% reported having ever had a cancer diagnosis (Table 2).

**Table 2:** Sample characteristics

Variable	Descriptive statistic	
	N*	%
*Age, years*		
40–49	18	18
50–59	20	21
> 60	60	61
*Sex*		
Male	34	35
Female	64	65
*Deprivation Quartile*		
Most deprived	56	57
Second most deprived	20	21
Second least deprived	19	19
Least deprived	3	3
*Employment status*		
Employed	21	21
Unemployed	27	28
Retired	50	51
*Highest level of education*		
No formal qualification	54	55
GCSE or equivalent	19	20
Higher education below degree level	16	16
Degree level or higher	7	7
Other	2	2
*Ethnic origin*		
Welsh/English/Scottish/ Northern Irish/ British	91	93
Bangladeshi	1	2
African	2	2
Caribbean	2	2
Other	2	1
*Cancer diagnosis*		
Yes	12	12
No	86	88

*N = 98, no missing data

### Secondary outcomes

#### Cancer symptom knowledge

Participants recognised on average one extra cancer symptom post intervention, with an average total symptom recognition score of 10 (score range: 0–14) at baseline and 11 (score range: 0–14) at follow up. As shown in Table 3, baseline ceiling effects were observed for recognition of specific cancer symptoms including lump (95%), rectal bleeding (94%) and a change in how skin looks (91%). The highest potential for improved symptom recognition was observed for unexplained change in appetite (41 to 65%), feeling bloated on most days (37 to 52%) and problems when peeing (50 to 66%).

**Table 3:** Cancer symptom recognition at baseline and one month follow-up

^b^Cancer symptoms (answered: ‘*yes’*)	Baseline descriptive statistic (n = 98)		One month descriptive statistic (*n* = 82^a^)	
	n	%	n	%
A cough that won’t go away	75	77	63	77
An unusual lump	93	95	73	89^1^
A change in how your skin looks	89	91	72	88
A sore or ulcer in your mouth that will not heal	68	70	65	79
A change in your poo	77	79	67	82
Blood in your poo	92	94	76	93
Problems when peeing	49	50	55	66^2^
Unexplained bleeding (e.g. blood in urine, rectal bleeding, vaginal bleeding during/after sex or in between periods)	83	85	72	88
Difficulty swallowing	63	64	59	72
Losing weight without trying to	85	87	66	81
Feeling bloated on most days	36	37	43	52^3^
An unexplained change in your appetite	40	41	53	65^4^
Feel tired most of the time	57	58	53	65^5^
An unexplained pain that won’t go away	73	75	61	75^6^

^a^Ns vary due to missing data for individual items in the cancer symptom recognition measure. Items where data were missing have been indicated (^1,3,6^ missing data for 2 participants, ^2,4,5^ missing data for 1 participant). One participant answered < 75% of the measure and was excluded from the analysis

^b^Wording of the symptoms reflect those used in the intervention

#### Anticipated symptom presentation

**Fig. 2:**
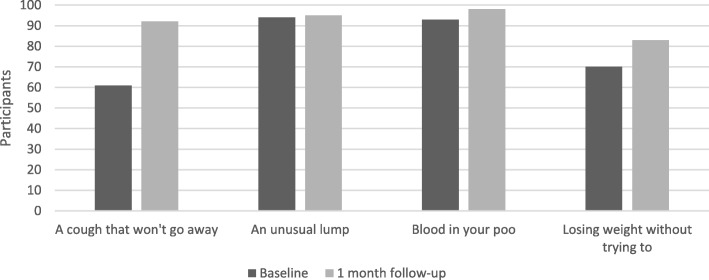
Anticipated time to symptom presentation: proportion stating that they would present within three weeks at baseline and follow-up

As depicted in Fig. 2, the largest improvement in anticipated time to symptom presentation was for persistent cough (32%), followed by unintended weight loss (14%). Anticipated symptom presentation increased by 6% for blood in stools and increased by 2% for an unusual lump.

#### State anxiety

The mean state anxiety score was 33.4 (SD = 12.6, range 20–80) at baseline and 34.3 (SD = 11.5, range 20–63) at one month follow-up. Scores at both time points were within the normal range [28].

#### Process measures of acceptability

Sixty-four participants (77%) at one month follow-up described the information in the health check as very useful, with 13 participants (16%) describing it as moderately useful and six (7%) as somewhat useful. Seventy-five participants (90%) thought the amount of information in the health check was about right, three (4%) thought there was too much, and five (6%) said there was not enough information. All 83 respondents (100%) said that they would recommend the health check to their family or friends.

### Qualitative interviews

Thirteen men and 12 women were interviewed, with a mean age of 66 years (range: 40–82). Interviews were on average 25 min long (range 14–43 min). Key themes included intervention acceptability and changes in symptom awareness and behaviour. Sample quotes are presented in Table 4 and referred to within the text in parentheses.

**Table 4:** Example quotes from process evaluation interviews

Coding	Example quotes	Text reference
Intervention acceptability	“Very personable, very approachable, a good listener. Took on board what I had to say, even though parts of it, because I talked about my Mum, I was quite upset.” Female, 48	A
	“It was more of a very friendly discussion about the areas that I could look at to improve, to give myself a better chance of surviving longer.” Male, 49	B
	“If there was a question that I wasn’t sure of, and it was sort of, say there was three different answers you could answer, and that answer wasn’t there, then I’d find it difficult, again I think that’s a generation thing, I’d rather verbally, rather than a screen or impersonal then put it that way.” Female, 63	C
	“It was understandable, easily understandable. It wasn’t difficult to understand and it was in plain English, which I thought was good.” Female 71	D
	“Personally I thought it was a little bit too much, to take in in one go, you just want to come out and come home, and said to my girls, I said well I can’t tell you, I wouldn’t have a clue. It’s too much to take in, there was a lot that I didn’t know, but I thought there was a lot to take in, again a little bit repetitive.” Female, 63	E
	“Some of the questions would be, not really concerned with, like this one ‘have you been losing weight without trying to?’ Yes or no, with me I’m on so many tablets, some months I put weight on, so it’s not difficult to answer it correctly but it’s a little bit of controversy, if you see what I mean?.” Female, 63	F
	“In my head I was thinking, I already know that, I want you to talk to me about the things that did flag up, to me that’s the important bit, I need to know more about that so I can change.” Male, 50	G
	“Very convenient. If I had to travel somewhere I don’t think I would have gone. Because it was here and I didn’t have to go out my way it was much easier.” Male, 50	H
Changes in symptom awareness and behaviour	“My daughter now will, instead of making chocolate sandwiches for work, I will do her a pasta salad and things like that so they love it, they love the change.” Female, 40	I
	“Let’s have a look at what I am eating, what I am drinking, what I am smoking. All the, what I am, what I thought was reasonable, some of them are not so reasonable, and I do need to back track and think. And I have.” Male, 49	J
	“I am cautious about myself, especially for example when I am changing from day clothes to evening pyjamas or when I am in the shower, I have a big mirror in my bathroom so I do tend to look over my body, so that shows me various things, and I reveal those to my GP when I go to see him” Male, 65	K
	“I didn’t really know that, if you had a persistent cough you should go and see about it because I would have thought it’s just sore throat or something.” Male, 56 years	L
	“I didn’t realise all the symptoms. It was informative, eye opening” Female, 40 years	M

#### Intervention acceptability

The rapport that was built between the lay advisor and the participant was an important aspect of intervention acceptability. Participants felt as though they were listened to and could therefore discuss sensitive topics during the health check (A). Participants’ overall views and experiences of doing the health check were positive and they found the content of the intervention engaging. Furthermore, participants reported that being approached in a community setting to talk about cancer was acceptable. It was felt that the friendly and informal nature of the intervention made for a pleasant, empowering experience and health check users enjoyed having the opportunity for focused discussion on their health (B).

A range of preferences were expressed for completion of the onscreen health check questionnaire. Older participants expressed a preference for the lay advisor to facilitate completion of health check. However, some participants felt confident in completing the health check on the iPad. Both methods of delivering the health check were found to be acceptable and the tailored nature of the intervention meant that either method could be easily implemented (C).

Participant feedback regarding the intervention content, such as the information presented and the language used, demonstrated that it was acceptable to users. Participants did not express any difficulties with understanding the information given to them during the health check and thought it was delivered in an easy to understand, user friendly manner (D). Some participants mentioned that the health check was slightly repetitive and that there was too much information (E).

Some symptom questions in the health check, such as feeling bloated and losing weight without trying to, were difficult for some participants to answer. This was mainly due to the older age of the sample and potential for comorbid health issues that gave rise to some participants experiencing symptoms included in the health check (F).

While the results section was generally acceptable to participants, some felt that exploring “green” results (where no action was suggested) was not as beneficial as discussing “amber” or “red” results (where action was indicated). These participants felt they already knew about certain cancer symptoms and that some risk factors were not relevant to them. They therefore considered that in-depth coverage of these areas was not beneficial (G).

Distance to travel to do the health check was generally considered by participants to be convenient and the ability to easily access the health check was described as important. Many of the health check locations were situated centrally within deprived communities and at a venue where potential participants would attend during the day (H).

#### Changes in symptom awareness and behaviour

Participants described making changes to their lifestyles since taking part in the health check, including improving their diet, engaging in more physical activity and decreasing alcohol consumption. The importance of social networks, such as family and friends, in supporting these changes was reported by participants. Receiving social support meant that participants were more motivated to continue eating a healthy diet and these changes could in turn positively impact family members (I).

The intervention made participants think more about their health in relation to their age and acted as a prompt to consider making changes to their diet, smoking habits and alcohol consumption (J). Many participants described the health check as an opportunity to identify areas of their health and lifestyle that they could improve.

Participants discussed the importance of checking for potential symptoms of cancer, and many participants reported carrying out health protective behaviours, such as checking for lumps in the shower, since taking part in the intervention. The ability to integrate these behaviours into a daily routine, such as showering or getting dressed, was mentioned and suggests that since doing the health check many participants found this behaviour change to be manageable and easy to adopt (K).

Participants reflected on new knowledge that they had obtained from the health check about presenting to their GP with vague cancer symptoms, such as a persistent cough. Before taking part in the intervention, participants discussed not having previous knowledge of these potential symptoms and that the intervention offered new information about the importance of presenting to the doctor (L, M).

## Discussion

To our knowledge the current study is the first to evaluate a community-based intervention designed to increase cancer awareness and encourage early symptom presentation among adults living in deprived communities. The current feasibility phase was an opportunity to explore contextual factors relating to recruitment settings and to enhance the intervention in preparation for a future trial of effectiveness. The health check intervention was found to be acceptable to participants and was feasible to deliver within community and healthcare settings, with evidence of reach to individuals from low socioeconomic groups.

We found strong support for the proposed theoretical mechanisms of change likely to positively influence cancer awareness and behavioural outcomes. During phase 1 of ABACus, the Behaviour Change Wheel [21] was used to guide the selection of intervention functions and content. One of the key functions that was identified during the developmental phase as being integral to the health check was environmental restructuring [19], with the present study providing evidence that this aspect was integral to the acceptability and feasibility of the intervention. The intervention restructured the physical and social environment through delivery in non-medical community settings, and through provision of social encouragement and support from a lay advisor who was able to build rapport with participants. Findings from the qualitative interviews confirmed that these mechanisms were practicable to implement and agreeable to participants, and that the lay advisor was perceived as a trusted source of information and advice about cancer awareness and lifestyle risks. These findings are promising, and support the recommendations of a systematic review [16] for interventions that target local communities as a way to encourage timely cancer symptom presentation among people from low socioeconomic groups. The health checks took place at convenient local community venues using opportunistic recruitment processes, thereby removing practical barriers to access such as difficulties with transport. Interview participants described the suitability of the health check locations.

The current research suggests that community-based recruitment methods are essential for engaging deprived populations in cancer awareness interventions. Although it was feasible to recruit in both community and healthcare settings, recruitment rates were higher in community venues such as health events, sheltered housing and food banks. A recent systematic review [29] found that facilitators for involving ‘hard to reach’ groups in health promotion interventions included the use of incentives and well-targeted community advertising. Although financial incentives were given for participation in the current study, the health check was not widely advertised. Employing highly proactive recruitment strategies in future research may help to further increase recruitment of the target population in healthcare settings.

The health check intervention was successful in reaching adults from low socioeconomic groups, with over half of the sample (55%) having no formal qualification. In addition, study retention exceeded expectations and is a further indicator of intervention feasibility and acceptability. As well as being an acceptable means of engaging participants, the health check was considered to be useful and easy to understand in terms of its information content. With a growing body of evidence suggesting that individuals with low health literacy are more likely to misunderstand health-related information [30], and potential for low health literacy in the target population, it was essential that the health check content was accessible and comprehensible. Participants were receptive to the information delivered in the health check, and described the importance of having the lay advisor present to empower and encourage them. However, intervention feedback from the qualitative interviews suggested that the duration of the health check and repetition of content were undesirable, indicating a need for content refinements to further enhance intervention acceptability.

The current feasibility study presented an opportunity to examine the change processes underpinning the health check, and therefore its potential to influence outcomes relating to cancer awareness in socioeconomically deprived groups. Evidence from the prospective questionnaire study indicated that the combination of intervention functions reflecting education, persuasion and enablement may encourage symptom awareness and motivate behaviour change [19]. As well as increasing knowledge and awareness, an important function of the health check is to counter fearful and fatalistic beliefs about cancer [11, 31] using persuasive and empowering messages delivered by a trained lay advisor. The inclusion of theory-derived mechanisms may therefore explain the potential for change that was observed for recognition of non-specific cancer symptoms and anticipated help-seeking behaviour in the current phase. Additionally, quantitative data indicated that all participants at one month follow-up had recommended the health check to their friends or family, demonstrating the potential for health check messages to harness the ‘lay system’ of health care [32] and to reach individuals within surrounding social circles. Public awareness of vague cancer symptoms is poor, especially among low socioeconomic groups [33] and therefore targeted community-based cancer awareness interventions should aim to raise awareness of vague, non-specific symptoms and tackle fear associated with going to the doctor, in order to encourage early presentation.

We acknowledge the limitations of conducting this feasibility study in one location (socioeconomically deprived areas of South Wales), and that the findings may not be generalisable to other geographical areas. Participants were recruited using opportunistic sampling methods, which may also limit representativeness. However, previous research has observed similar levels of cancer knowledge, beliefs and barriers across Wales, England and Northern Ireland [34], hence the current findings may be applicable and the intervention itself transferable to other deprived areas of the UK. The effectiveness of the health check in increasing cancer awareness and help-seeking behaviour should therefore be tested in the context of a multi-centre controlled trial. In addition, although the ABC measure has been internationally validated [27], its validity in the context of socioeconomic deprivation is unclear and future research should aim to psychometrically test the adapted ABC measure for use with individuals from lower socioeconomic groups.

## Conclusion

The current research generated evidence about theory-based mechanisms of change that are likely to overcome barriers to cancer awareness and help-seeking behaviour in deprived populations. Intervention recruitment methods were feasible, especially in non-medical community settings, and the facilitated health check reached adults from low socioeconomic groups. Prospective findings indicated the health check’s potential to improve cancer awareness among adults living in deprived communities, which should be tested in the context of a controlled trial of effectiveness. Longer-term, the health check may be implemented to empower communities most affected by cancer, and may contribute to reducing socioeconomic inequalities in cancer survival outcomes in the UK.

## Additional files

Additional file 1: National Awareness and Early Diagnosis Initiative (NAEDI). This file further describes the influence of socioeconomic status on cancer survival and premature mortality in the United Kingdom. (PDF 124 kb)

Additional file 2: Intervention questions. This file details all questions that participants completed during the health check intervention. (DOCX 15 kb)

## Footnotes

1 Increasing knowledge or understanding

2 Increasing means and reducing barriers to increase capability (beyond education or training) or opportunity (beyond environmental restructuring)

3 Using communication to induce positive or negative feelings to stimulate action

4 Changing the physical or social context

## References

[1] Mastrangelo G (2013). Endotoxin and Cancer chemoprevention. Cancer Epidemiol.

[2] Maddams J, Brewster D, Gavin A, Steward J, Elliott J, Utley M, Møller H (2009). Cancer prevalence in the United Kingdom: estimates for 2008. Br J Cancer.

[3] Cancer Research UK (2015a) Cancer survival statistics[online] Available at: http://www.cancerresearchuk.org/health-professional/cancer-statistics/survival. Accessed 4 Dec 2017.

[4] Sant M, Allemani C, Santaquilani M, Knijn A, Marchesi F, Capocaccia R (2009). Eurocare working group. Survival of cancer patients diagnosed in 1995-1999. Results and commentary. Eur J Cancer.

[5] Berrino FV, Lutz A, Lombardo J, Micheli C, Capocaccia A (2009). EUROCARE working group. Comparative cancer survival information in Europe. Eur J Cancer.

[6] Verdecchia A, Guzzinati S, Francisci S, DeAngelis R, Bray F, Allemani C, Tavilla A (2009). Survival trends in European cancer patients diagnosed from 1988 to 1999. Eur J Cancer.

[7] Foot C, Harrison T (2011) How to improve cancer survival: Explaining England's relatively poor rates. [PDF document] Available at: https://www.kingsfund.org.uk/sites/files/kf/How-to-improve-cancer-survival-Explaining-England-poor-rates-Kings-Fund-June-2011.pdf. Accessed 4 Dec 2016.

[8] Cancer Research UK (CRUK). *Cancer and health inequalities: an introduction to current evidence* (2006). Retrieved from http://www.cancerresearchuk.org/prod_consump/groups/cr_common/@nre/@pol/documents/generalcontent/crukmig_1000ast-3344.pdf

[9] Rachet B, Ellis L, Maringe C (2010). Socioeconomic inequalities in cancer survival in England after the NHS cancer plan. Br J Cancer.

[10] Lyratzopoulos G, Abel G, Brown C (2013). Socio-demographic inequalities in stage of cancer diagnosis: evidence from patients with female breast, lung, colon, rectal, prostate, renal, bladder, melanoma, ovarian and endometrial cancer. Ann Oncol.

[11] Whitaker K, Scott S, Wardle J (2015). Applying symptom appraisal models to understand sociodemographic differences in responses to possible cancer symptoms: a research agenda. Br J Cancer.

[12] Lyratzopoulos G, Saunders C, Abel G, McPhail S, Neal R, Wardle J, Rubin G (2015). The relative length of the patient and the primary care interval in patients with 28 common and rarer cancers. Br J Cancer.

[13] Macleod U, Mitchell E, Burgess C, Macdonald S, Ramirez A (2009). Risk factors for delayed presentation and referral of symptomatic cancer: evidence for common cancers. Br J Cancer.

[14] Richards MA (2009). The national awareness and early diagnosis initiative in England: assembling the evidence. Br J Cancer.

[15] Chatwin J, Povey A, Kennedy A (2014). The mediation of social influences on smoking cessation and awareness of the early signs of lung cancer. BMC Public Health.

[16] McCutchan GM, Wood F, Edwards A, Richards R, Brain KE. Influences of cancer symptom knowledge, beliefs and barriers on cancer symptom presentation in relation to socioeconomic deprivation: a systematic review. BMC Cancer. 2015;15(1) 10.1186/s12885-015-1972-8 PMC4688960 26698112

[17] Smith LK, Pope C, Botha JL (2005). Patients' help-seeking experiences and delay in cancer presentation: a qualitative synthesis. Lancet.

[18] Walter F, Webster A, Scott S, Emery J (2012). The Anderson model of Total patient delay: a systematic review of its applications in cancer diagnosis. J Health Serv Res Policy.

[19] Smits S, McCutchan G, Wood F, Edwards A, Lewis I, Robling M, et al. Development of a behavior change intervention to encourage timely Cancer symptom presentation among people living in deprived communities using the behavior change wheel. Ann Behav Med. 2016:1–15. 10.1007/s12160-016-9849-x. 10.1007/s12160-016-9849-x PMC6367899 27826697

[20] Craig P, Dieppe P, Macintyre S, et al. Developing and evaluating complex interventions: the new Medical Research Council guidance. BMJ. 2008;337:a1655–5. 10.1136/bmj.a1655 PMC2769032 18824488

[21] Michie MS, West R. The behaviour change wheel: a new method for characterising and designing behaviour change interventions. BMC Implementation Science. 2011;6(42) 10.1186/1748-5908-6-42 PMC3096582 21513547

[22] Michie S, Richardson M, Johnston M (2013). The behavior change technique taxonomy (v1) of 93 hierarchically clustered techniques: building an international consensus for the reporting of behavior change interventions. Ann Behav Med.

[23] UK Government Alcohol Consumption: Advice on Low Risk Drinking (2011). Retrieved from https://www.gov.uk/government/publications/alcohol-consumption-advice-on-low-risk-drinking

[24] UK Government Physical Activity Guidelines: Department of Health and Social Care (2011). Retrieved from https://www.gov.uk/government/publications/uk-physical-activity-guidelines

[25] NHS Choices: BMI Healthy Weight Calculator [online] Available at: https://www.nhs.uk/Tools/Pages/Healthyweightcalculator.aspx. Accessed 4 Dec 2017.

[26] NHS Choices: 5 A Day [online] Available at: https://www.nhs.uk/LiveWell/5ADAY/Pages/5ADAYhome.aspx. Accessed 4 Dec 2017.

[27] Simon AE, Forbes LJL, Boniface D, Warburton F, Brain KE, Dessaix A, et al. An international measure of awareness and beliefs about cancer: development and testing of the ABC. BMJ Open. 2012;2(6) 10.1136/bmjopen-2012-001758 PMC3547316 23253874

[28] Marteau T, Bekker H (1992). The development of a six-item short-form of the state scale of the Spielberger state-trait anxiety inventory (STAI). Br J Psychol.

[29] Liljas AEM, Walters K, Jovicic A, Illiffe S, Manthorpe J, Goodman C, Kharicha K (2017). Strategies to improve engagement of ‘hard to reach’ older people in research on health promotion: a systematic review. BMC Public Health.

[30] Friedman DB, Hoffman-Goetz L, Arocha JF (2006). Health literacy and the world wide web: comparing the readability of leading incident cancers on the internet. Medical Informatics and the Internet in Medicine.

[31] McCutchan GM, Wood F, Edwards A, Richards R, Brain KE (2016). Barriers to cancer symptom presentation among people from low socioeconomic groups: a qualitative study. BMC Public Health.

[32] Pescosolido B, Boyer C, Scheid T, Brown T (1999). How do people come to use mental health services? Current knowledge and changing perspectives pp 392–411 In A Handbook for the study of mental health: Social contexts, theories and systems.

[33] Quaife S, Forbes L, Ramirez A (2013). Recognition of cancer warning signs and anticipated delay in help-seeking in a population sample of adults in the UK. Br J Cancer.

[34] Forbes LJL, Warburton F, Richards MA, Ramirez AJ (2014). Risk factors for delay in symptomatic presentation: a survey of cancer patients. Br J Cancer.

