# Supplementary material for: Feasibility and acceptability of a cancer symptom awareness intervention for adults living in socioeconomically deprived communities
Source: BMC Public Health. 2018 Jun 5;18:695. doi: 10.1186/s12889-018-5606-3 (PMC5989371; doi:10.1186/s12889-018-5606-3)
Supplement: Supplementary file 1 — National Awareness and Early Diagnosis Initiative (NAEDI). This file further describes the influence of socioeconomic status on cancer survival and premature mortality in the United Kingdom. (PDF 124 kb) [file 12889_2018_5606_MOESM1_ESM.pdf]

National Awareness and Early Diagnosis Initiative (NAEDI)

NAEDI formally launched in 2008 and continues as a partnership between public and third sector organisations. Its role is to provide leadership and support to activities and research that promote earlier diagnosis of cancer.

Why take action to diagnose cancer earlier?

When cancer is diagnosed at an early stage, treatment options and chances of a full recovery are greater.

For example, over 93% of bowel cancer patients diagnosed with the earliest stage of disease survive at least five years compared with less than 7% of those diagnosed with the most advanced stage disease.

The same pattern is true for lung cancer, breast cancer, and for many cancers, common or rare.

We know that inequalities exist, with some groups of patients more likely to be diagnosed with later stage disease.

By prompting, coordinating and supporting early diagnosis activity, NAEDI aims to make a difference to the lives of thousands of cancer patients.

Is England really any worse than other countries when it comes to diagnosing cancers early?

Various studies comparing cancer survival across countries have shown that England does worse than comparable countries. With the exception of breast cancer, we don't seem to be narrowing the gap on survival when looking at patients diagnosed up to 2007.

There are many factors which influence a patient's chances of surviving cancer, not least the treatment they receive. But another major factor is the stage of the disease at diagnosis and, for some cancers at least, there's evidence that more patients in England are diagnosed at a later stage compared with other countries.

Even for those cancers where, overall, our stage distribution compares favourably internationally, there's still unacceptable variation within England and no doubt that diagnosis at an earlier stage is associated with better outcomes.

The drive to improve early diagnosis of cancer in England continues.

Where do we need to take action?

As with survival, there are likely to be many factors which affect the stage of disease at diagnosis and we need to tackle each element of potential delay.

This means raising awareness of what to look out for and when to act; tackling negative attitudes to cancer and barriers to seeing the doctor amongst the public; supporting primary care so they are able to manage and refer patients with symptoms that might be cancer appropriately; and ensuring optimum and prompt access to diagnostic tests and referral pathways that can facilitate the diagnosis of cancer.

Public health and the NHS both have a role to play in tackling late diagnosis and their progress will be measured through indicators in their Outcome Frameworks.

To bring about real change in diagnosing cancers earlier, action is needed across the whole pathway.

What if the patient pathway doesn't go to plan?

If a patient presents to their doctor with symptoms that the GP suspects could be cancer, the patient receives an urgent referral to secondary care and is seen within two weeks (two week wait).

But when the pathway to the diagnosis of cancer has been studied, it's clear that many patients with cancer don't come through this route. Routes to Diagnosis analysis highlighted that almost a quarter of newly diagnosed cancer patients came through as emergency presentations.

The concerning news here is that one-year survival rates for these patients was much lower than for those coming through other routes. The study also shows variation in emergency presentations by cancer type, age and deprivation.

Activity is underway to find out more about the patients who present as emergencies and to unpick where any delays might have occurred.

It's clear that NAEDI's aim to promote earlier diagnosis, working across the patient pathway, is as important as ever if we are to prevent avoidable deaths from cancer.

Useful links:

www.naedi.org.uk
www.icbp.org.uk
www.rcgp.org.uk/clinical-and-research/clinical-resources/cancer
www.ncin.org.uk/publications/routes_to_diagnosis

NAEDI is a public sector/third sector partnership. It is led by NHS England, Department of Health, Public Health England and Cancer Research UK. They work in partnership with other public and voluntary sector organisations to support and drive forward work on early diagnosis.
