# Supplementary material for: Feasibility and acceptability of a cancer symptom awareness intervention for adults living in socioeconomically deprived communities
Source: BMC Public Health. 2018 Jun 5;18:695. doi: 10.1186/s12889-018-5606-3 (PMC5989371; doi:10.1186/s12889-018-5606-3)
Supplement: Supplementary file 2 — Intervention questions. This file details all questions that participants completed during the health check intervention. (DOCX 15 kb) [file 12889_2018_5606_MOESM2_ESM.docx]

**Supplementary file 2.** Intervention questions

| **Intervention domain** | **Questions** |
| --- | --- |
| *‘Your health’* | 1. *Do you have a cough that won’t go away?* 2. *Have you noticed any unusual lumps on your body (e.g. breasts, testicles, armpits, groin)?* 3. *Have you noticed a change in how your skin looks (a change to a mole, freckle or patch of skin)?* 4. *Do you have a sore or ulcer in your mouth that will not heal?* 5. *Have you noticed a change in your poo?* 6. *Have you noticed any blood in your poo?* 7. *Do you have any problems when peeing?* 8. *Do you have any unexplained bleeding (e.g. blood in your pee, bleeding from your bottom, vaginal bleeding during/after sex or in between periods)?* 9. *Do you have any difficulty swallowing?* 10. *Have you been losing weight without trying to?* 11. *On most days, do you feel bloated?* 12. *Have you noticed any unexplained change in your appetite?* 13. *Do you feel tired most of the time?* 14. *Do you have an unexplained pain that won’t go away?* |
| *‘About you’* | 1. *Have you ever been diagnosed with cancer?* 2. *Are there two or more close relatives on the same side of your family who have had cancer?* 3. *Do you have any relatives who have been under the age of 50 when diagnosed with cancer?* 4. Body Mass Index (BMI) is a good way of measuring if you’re a healthy weight for your height. Click on Imperial or Metric to add information about your height and weight. 5. *Please give your height in feet and inches* 6. *Please give your weight in stones and pounds* 7. *Have you ever been invited for a cervical smear test? (female only)* 8. *Have you been invited for your breast screening by Breast Test Wales? (female only)* 9. *Did you receive your bowel screening kit in the post? (male and female)* |
| *‘Your lifestyle’* | 1. *Do you smoke? [If yes- On average how many cigarettes a day do you smoke?]* 2. *Are you exposed to another person’s smoke on a regular basis?* 3. *Do you drink alcohol? [If yes- On average how many units of alcohol do you drink each week?]* 4. *On average how many days a week do you exercise for half an hour or more?* 5. *How often do you eat 5 portions of fruit and vegetables?* |
